# Examining the Impacts of Urban Form on Air Pollution in Developing Countries: A Case Study of China’s Megacities

**DOI:** 10.3390/ijerph15081565

**Published:** 2018-07-24

**Authors:** Chunshan Zhou, Shijie Li, Shaojian Wang

**Affiliations:** Guangdong Provincial Key Laboratory of Urbanization and Geo-simulation, School of Geography and Planning, Sun Yat-sen University, Guangzhou 510275, China; zhoucs@mail.sysu.edu.cn (C.Z.); lishij2@mail2.sysu.edu.cn (S.L.)

**Keywords:** urban form, air pollution, landscape metrics, panel data analysis

## Abstract

Urban form is increasingly being identified as an important determinant of air pollution in developed countries. However, the effect of urban form on air pollution in developing countries has not been adequately addressed in the literature to date, which points to an evident omission in current literature. In order to fill this gap, this study was designed to estimate the impacts of urban form on air pollution for a panel made up of China’s five most rapidly developing megacities (Beijing, Tianjin, Shanghai, Chongqing, and Guangzhou) using time series data from 2000 to 2012. Using the official Air Pollution Index (API) data, this study developed three quantitative indicators: mean air pollution index (MAPI), air pollution ratio (APR), and continuous air pollution ratio (CAPR), to evaluate air pollution levels. Moreover, seven landscape metrics were calculated for the assessment of urban form based on three aspects (urban size, urban shape irregularity, and urban fragmentation) using remote sensing data. Panel data models were subsequently employed to quantify the links between urban form and air pollution. The empirical results demonstrate that urban expansion surprisingly helps to reduce air pollution. The substitution of clean energy for dirty energy that results from urbanization in China offers a possible explanation for this finding. Furthermore, urban shape irregularity positively correlated with the number of days with polluted air conditions, a result could be explained in terms of the influence of urban geometry on traffic congestion in Chinese cities. In addition, a negative association was identified between urban fragmentation and the number of continuous days of air pollution, indicating that polycentric urban forms should be adopted in order to shorten continuous pollution processes. If serious about achieving the meaningful alleviation of air pollution, decision makers and urban planners should take urban form into account when developing sustainable cities in developing countries like China.

## 1. Introduction

Human activity is the main driving factor intensifying a range of environmental issues [1]. The concentration of people and economic activities in emerging megacities has been accompanied by a growing vehicle fleet [2], a development that has led to rapid increases in energy consumption, exhaust emissions, and consequently air pollution, especially in developing countries [3]. There is no doubt that ambient air pollution, which constitutes a pressing environmental concern, poses a range of negative health implications at local, regional, and global scales [4], including higher instances of cardiorespiratory diseases among urban dwellers [5]. These impacts incur real costs for individuals, medical systems, and economies [6]. Due to the inevitability and intractability of such effects, much scholarly attention has been directed towards identifying the influencing factors behind air pollution. The role played by the spatial configuration and urban form of cities in tackling environmental pressure is increasingly being recognized by the academic community [7,8]. Specifically, this paper addressed the extent to which spatial optimization and urban planning could either improve or worsen the urban environment, by specifically investigating the impacts of urban form on air pollution, which is of great significance for alleviating pollution and achieving sustainable development.

Many previous studies addressed the mechanisms by which urban form might influence air pollution, both theoretically [7,9,10] and empirically [11,12,13,14,15,16,17,18]. From the theoretical perspective, air pollution is believed to maintain a high correlation with energy consumption in terms of both industrial processes and vehicles, as the combustion of fossil fuel is the primary source of many important pollutants, such as sulfur dioxide (SO_2_) and carbon monoxide (CO) [7]. Whilst urban form is less likely to affect the air pollution generated by industrial emission sources (which maintain relatively stable locations), it does exert clear effects on the air pollution associated with automobiles, by influencing urban traffic patterns and citizens’ travel behavior [9]. Generally speaking, compact (that is mixed-use and high-density) urban form is negatively correlated with auto dependence and positively correlated with the use of public transit and walking, and thus the mitigation of air pollution [10]. The correlation between urban form and air pollution have been estimated empirically using a variety of methodologies, particularly in relation to developed countries [11]. In general, two kinds of measurements are commonly utilized to quantify air pollution: (i) the Air Quality Index (AQI) and (ii) the concentration of certain pollutants, such as nitrogen dioxide (NO_2_), fine particulate matter (PM_2.5_), and ozone (O_3_). McCarty and Kaza investigated the associations between several urban landscape metrics and AQI in the U.S., associating sprawling and fragmentary urban spatial structure with lower air quality [12]. Stone [13] estimated the association between urban form and O_3_ levels in 45 large U.S. metropolitan areas, finding that excessive O_3_ levels were more likely to occur in decentralized metropolitan regions than in spatially compact metropolitan regions. Similar conclusions were drawn by Schweitzer and Zhou in their study of neighborhood-level O_3_ concentrations in 80 U.S. metropolitan regions [14]. Focusing on the New York City (NYC) metropolitan region in the U.S., Civerolo et al., modeled the potential effects of urban expansion on O_3_ concentrations, concluding that extensive changes in urban land cover may result in higher O_3_ concentrations [15]. Mansfield et al., [16] and Hixson et al., [17] have both investigated the impacts of different development scenarios on PM_2.5_ concentrations, coming to the same conclusion that compact development reduced these concentrations, while sprawling development increased them. A study by Bechle et al., quantified the effects of urban form on NO_2_ concentrations for 83 cities globally, finding a negative correlation between urban contiguity and NO_2_ concentrations, and no statistically significant correlation between urban compactness and NO_2_ concentrations [18]. In contrast, Borrego et al., concluded that compact urban morphology with mixed land uses helped to lower NO_2_ concentrations [9].

In spite of abundant studies on the correlation between urban form and air pollution in developed countries, a limited amount of literature has addressed this significant issue in the context of developing countries, especially China. Due to unprecedented industrialization and urbanization in recent decades, China has become the largest developing country in the world [19], an achievement that has been accompanied by substantial environmental deterioration, especially air pollution [6]. Urban areas constitute the major contributor to China’s economic growth, and consume more than half of the country’s energy [20]. Chinese residents, particularly urban residents, are increasingly responding to air pollution [21], making ambient air pollution one of the top environmental issues in China [22]. Improving air quality is now recognized as being critical to achieving long-term sustainability in China. Therefore, the Chinese government needs not only to foster continued economic growth, but also to curb air pollution [23]. Existing studies on air pollution in China have engaged in the identification of air pollution sources [24], as well as the spatiotemporal variations of air pollution [25,26]. Moreover, the factors influencing air pollution in China, including meteorological factors, socioeconomic factors, and policy factors, have been identified by a number of scholars. Meteorological factors, such as atmospheric pressure [27], wind speed [28], temperature [29], relative humidity [30], and breeze circulation [31], have all been identified as fundamental determinants of air pollution in China. Aside from these meteorological factors, it is commonly known that air pollution is strongly influenced by human activities, and socioeconomic factors have been proven to maintain a high correlation with air pollution in China, a correlation that reflects the country’s recent economic prosperity. For example, applying the Environmental Kuznets model, Poon et al. investigated the effects of a number of economic indicators addressing energy, transport, and trade on China’s air pollution levels [32]. Kim et al. utilized city-level panel data and spatial econometric models to examine the socioeconomic factors that influence air pollution in Chinese cities, including industrial structure, population density, green spaces, and public transit [33]. Using spatial regression and geographical detector technique, Zhou et al., estimated the impacts of socioeconomic factors on PM_2.5_ in China’s cities [34]. In addition, it might also be noted that the connections between China’s air pollution and several other specific factors have been investigated, such as winter heating [35], ship emissions [36], and Chinese Nian culture [37]. Furthermore, scientists have also taken an interest in the effectiveness of a range of policies implemented by the Chinese government in order to alleviate air pollution, such as vehicle emissions standards [38], energy saving and emission reduction regulations [39], and a range of measures to ensure haze-free skies during the 22nd Asia-Pacific Economic Cooperation (APEC) conference [40]. However, the impact of urban form on air pollution in China has not been adequately addressed in studies to date, which is taken as an evident omission within the existing literature.

While the correlation between urban form and air pollution has been widely demonstrated in the context of developed countries, there has been limited progress in bringing urban form into the sphere of factors that are understood to influence air pollution in China or highlight the potential role that urban planning and spatial optimization might play in controlling air pollution. Addressing this gap, this case study of China’s five most rapidly developing megacities was designed in order to contribute to scholarly understanding of the mechanisms by which urban form might affect air pollution in the context of developing countries. Three indicators based on the Air Pollution Index (API) were first developed in order to evaluate air pollution levels. These formed the dependent variables of the study. Urban form was then quantified, which constituted the independent variables of the study, using a range of landscape metrics and remote sensing data. After completing these calculations, panel data models were implemented in order to estimate the degree of correlation between urban form and air pollution. The findings obtained from this study provide important decision support for decision makers and urban planners in controlling air pollution and building sustainable cities in China.

## 2. Study Area

**Figure 1:**
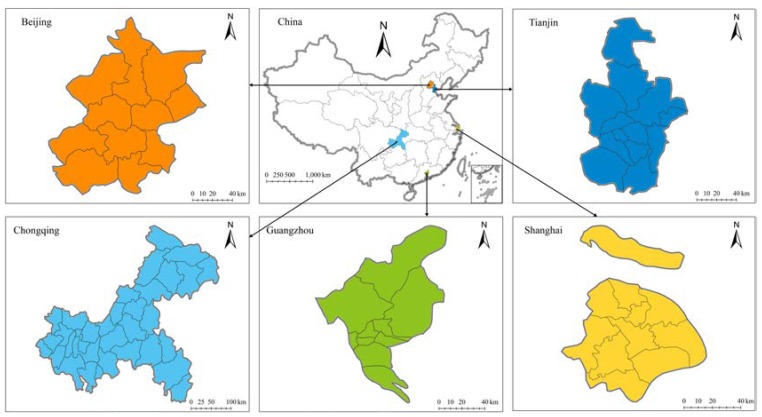
Locations of the five Chinese megacities in this study: Beijing and Tianjin in North China, Shanghai in East China, Chongqing in West China, and Guangzhou in South China.

Given the substantial relationship between air pollution and urban areas, this study selected five representative megacities in China as the study area: Beijing, Tianjin, Shanghai, Chongqing, and Guangzhou. As shown in Figure 1, these five megacities are situated in northern, eastern, western, and southern China. Beijing, China’s capital, is the country’s national center of politics and culture. Abutting Beijing and the Bohai Gulf, Tianjin is the largest coastal city in North China. Shanghai, which is situated in the Yangtze River Delta, is the economic, financial, and innovation center of China. As the only municipality in West China, Chongqing is the regional economic and transportation center for the upper Yangtze River. Guangzhou, located in the Pearl River Delta, is the provincial capital of Guangdong and the largest city in South China. An important reason for selecting these five megacities is that they are not only the largest but also the most developed cities in the country. Four of these cities are municipalities; the exception, Guangzhou, is the largest city in South China. The rapid industrialization and urbanization of these five megacities has led to environmental disruption, including air pollution, which poses a serious challenge for their sustainability. Far better understanding of the correlation between urban form and air pollution is required if policy makers are to be able to promote environment protection and air quality improvement.

## 3. Data and Method

### 3.1. Air Pollution Measurement and Data Processing

The Air Pollution Index (API) is a model that is commonly used to gauge the level of ambient air pollution. It takes the form of a non-dimensional number in a range of 0–500 [41] and offers a more generalized and simple way for the public to understand the degree of air pollution and the accompanying health effects [42]. Its statistical characteristics are described in Table 1. Daily API data for the five Chinese megacities studied, from 2000 to 2012, were derived from the data center of China’s Ministry of Environmental Protection (MEP) (http://datacenter.mep.gov.cn).

The API is an integrated measurement reflecting the levels of three fundamental atmospheric pollutants—namely, SO_2_, NO_2_, and suspended particulates (PM_10_), each of which are computed individually, with the highest API of the three air pollutants then being reported as the city’s API, and the corresponding pollutant identified as the “main pollutant” [43]. A method of linear interpolation was used to calculate API [44], specified as:

(1) $$API_{i}=\frac{\left(API_{U}-API_{L}\right)}{\left(C_{U}-C_{L}\right)\times \left(C_{i}-C_{L}\right)}+API_{L},$$

(2) $$\;API=\max\left(API_{i}\right),$$

where *API_i_* is the index for pollutant *i* (SO_2_, NO_2_, and PM_10_). A daily index is calculated for each air pollutant. *C_i_* is the observed concentration of pollutant *i*. *C_U_* and *C_L_* are the upper and lower limits of the interval (shown in Table 2), within which lies the *C_i_*. *API_U_* and *API_L_* are the upper and lower limits of the corresponding API interval. The API is defined as the maximum of *API_i_*, and the pollutant responsible for the highest index is the “main pollutant,” if the API is above 50.

On the basis of the daily API data, three derived indexes were further developed in order to assess annual air pollution levels in the five Chinese megacities: mean API (MAPI), air pollution ratio (APR), and continuous air pollution ratio (CAPR). 

MAPI is the average value of daily API and reflects the overall air pollution level for a whole year, a measure which overlooks the daily differences in air pollution and is susceptible to extreme values. According to Table 1, a day would be identified as “polluted” if it had an API that was greater than 100, and further defined “continuous air pollution” as a process defined by three or more uninterrupted air pollution days. As such, APR is measured by the proportion of the number of air pollution days in a year and represents the actual air pollution days with detrimental health effects. It is believed that continuous air pollution processes have greater adverse health effects than intermittent air pollution processes when controlling for the number of air pollution days. Taking the persistence and dispersal of air pollution into account, CAPR denotes the continuous air pollution process, which is measured by the ratio of the total amount of air pollution days in a continuous air pollution process to the total observed days in a year. The specified equations are as follows.

(3) $$\mathrm{MAPI}=\frac{\sum_{i=1}^{n}API_{i}}{N_{o}},$$

(4) $$\mathrm{APR}=\frac{N_{p}}{N_{o}},$$

(5) $$\mathrm{CAPR}=\frac{N_{cp}}{N_{o}},$$

where MAPI, APR, and CAPR denote API, air pollution ratio, and continuous air pollution ratio, respectively. *API_i_*indicates the air pollution index in *i*th day. *N_o_* represents the total observed days in a year. *N_p_* denotes the total amount of air pollution days. *N_cp_* represents the total amount of air pollution days in continuous air pollution process.

### 3.2. Remote Sensing Data and Landscape Metrics for Quantifying Urban Form

**Figure 2:**
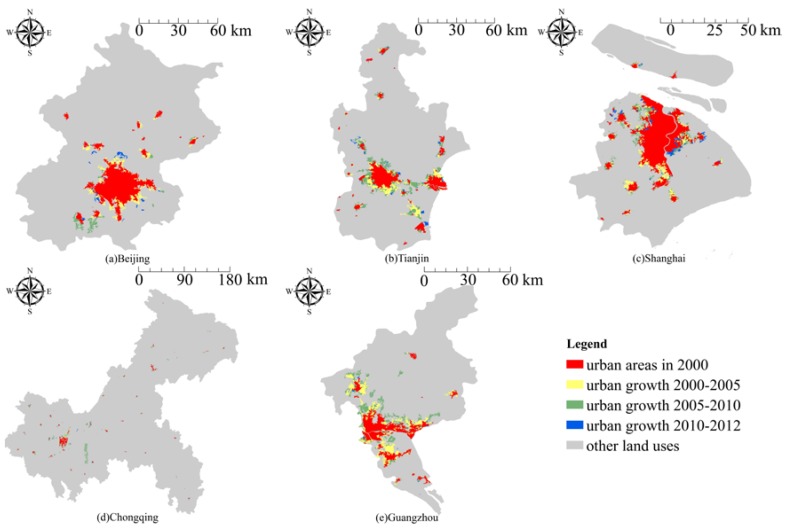
Changes in urban built-up areas between the years 2000–2012 in (**a**) Beijing; (**b**) Tianjin; (**c**) Shanghai; (**d**) Chongqing; and (**e**) Guangzhou.

Using land-use data from Landsat Enhanced Thematic Mapper (ETM) and Landsat Thematic Mapper (TM) scenes with a spatial resolution of 30 m × 30 m for four periods (2000, 2005, 2010, and 2012), which was developed by the Institute of Geographical Sciences and Natural Resources Research (IGSNRR, http://www.igsnrr.ac.cn) at the Chinese Academy of Sciences (CAS), urban built-up areas for each of the five megacities were extracted. Detailed extraction processes can be seen in relevant literatures [45,46]. Figure 2 presents the spatial patterns of urban growth in each of the megacities from 2000 to 2012.

Landscape metrics characterize the spatial configuration of a landscape [47], and have proved effective means for quantifying urban form [48,49]. Based on existing literature addressing urban form [50,51,52,53], seven common landscape metrics were selected to measure urban form in this study: total area (TA), perimeter-area fractal dimension (PAFRAC), area-weighted mean fractal dimension index (AWMFDI), mean perimeter-area ratio (MPARA), patch density (PD), landscape division index (DIVISION), and splitting index (SPLIT). These indicators represent three aspects of urban form—namely, urban size, urban shape irregularity, and urban fragmentation. Total area (TA) is a fundamental indicator for calculating a range of landscape metrics, and denotes the overall size of an urban area. Urban shape irregularity, which is interpreted as the degree to which the geometry of urban patches is considered to be “convoluted,” is represented by PAFRAC, AWMFDI, and MPARA. In general, the higher the values of PAFRAC, AWMFDI, and MPARA, the more complex the shape of the urban area. PD, DIVISION, and SPLIT assess the degree to which urban patches are scattered, and the lower the values of these three landscape metrics, the more compact the urban form is. All the landscape metrics applied in this research were calculated using the FRAGSTATAS 4.2 software, and Table 3 sets out the specific mathematical equations and descriptions of them.

### 3.3. Panel Data Analysis

Generally, the formulas of panel data models can be categorized into three types: the fixed coefficients and intercepts model, the constant coefficients and variable intercepts model, and the variable coefficients and variable intercepts model [54]. The fixed coefficients and intercepts model has constant slopes and intercepts, which can be expressed as Equation (6). As shown in Equation (7), the constant coefficients and variable intercepts model has constant slopes and variable intercepts. Equation (8) represents the variable coefficients and variable intercepts model, which has differential slopes and intercepts that vary based on time and/or entity.

(6) $$y_{it}=\alpha +\beta x_{it}+\varepsilon_{it},$$

(7) $$y_{it}=\alpha_{i}+\beta x_{it}+\varepsilon_{it},$$

(8) $$y_{it}=\alpha_{i}+\beta_{i}x_{it}+\varepsilon_{it},$$

where *i* is the number of observations over time; *t* denotes the time; *α_i_* is the intercept for discerning the fixed effects or random effects; and *ε_it_* is the error term.

An *F*-test, based on two main hypotheses (*H*_1_ and *H*_2_), is usually used in selecting the specific form of panel data model, which compares the residual sum of squares (RSS) of Equations (6)–(8). Given the confidence level, if *F*_2_ is larger than the critical value, the hypothesis *H*_2_ is accepted and Equation (6) is chosen; otherwise, the hypothesis *H*_1_ must be tested. If *F*_1_ is larger than the critical value, the hypothesis *H*_1_ is then accepted, and Equation (7) is chosen; otherwise Equation (8) is chosen.

(9) $$\;H_{1}:\beta_{1}=\beta_{2}=\cdots =\beta_{N}\;F_{1}=\frac{\left(S_{2}-S_{1}\right)/\left[\left(N-1\right)k\right]}{S_{1}/\left(NT-N\left(k+1\right)\right)}\sim F\left[\left(N-1\right)k\right],N\left(T-k-1\right)$$

(10) $$H_{2}:\alpha_{1}=\alpha_{2}=\cdots =\alpha_{N}\;\;\;\;\beta_{1}=\beta_{2}=\cdots =\beta_{N}\;F_{2}=\frac{\left(S_{3}-S_{1}\right)/\left[\left(N-1\right)\left(k+1\right)\right]}{S_{1}/\left(NT-N\left(k+1\right)\right)}\sim F\left[\left(N-1\right)\left(K+1\right),N\left(T-K-1\right)\right]$$

where *F*_1_ is the statistic under the hypotheses of *H*_1_ in which intercepts and slopes are constant over time; *F*_2_ is the statistic under the hypotheses of *H*_1_ in which intercepts are variable and slopes are constant over time; *S*_1_, *S*_2_, and *S*_3_ are RSS for Equations (6)–(8), respectively; and *N*, *T*, and *k* denote the number of observations over time, the number of years, and the number of independent variables, respectively.

After determining the specific form of the panel data model, whether the fixed effects estimator or the random effects estimator should be utilized needed to be decided. From a methodological perspective, allowing for the insertion of dummy variables, the fixed effects estimator can estimate parameters for panel models which have different intercepts for different time series or sections. The random effects estimator should be used if the intercept term in the fixed effects estimator contains the average effects of time series random error term and cross-sectional random error term. Generally, a Hausman test is employed to select the appropriate type of estimator [55].

## 4. Findings and Discussions

### 4.1. Evolution of Air Pollution, 2000–2012

**Figure 3:**
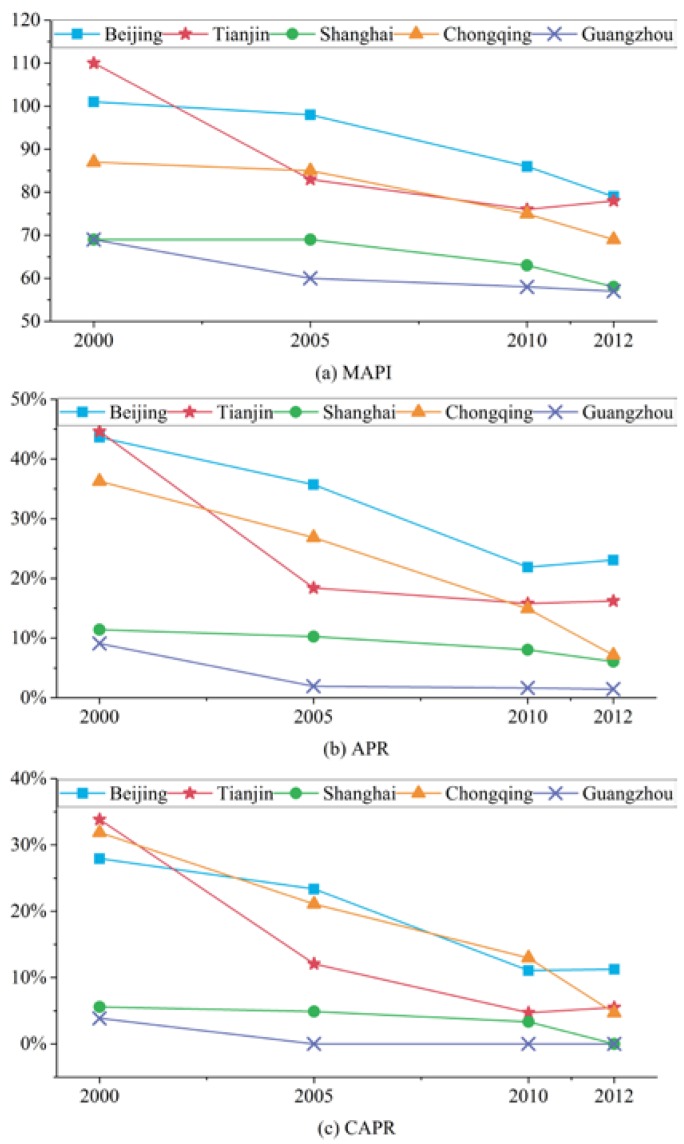
Air pollution in five Chinese megacities: (**a**) MAPI; (**b**) APR; and (**c**) CAPR, 2000–2012.

Figure 3a shows that MAPI generally decreased between 2000 and 2012, implying that the overall air quality of the five cities improved over this period. Tianjin evidences the most distinct reduction in MAPI, which decreased from 110 in 2000 to 78 in 2012. From Figure 3b, a downward trend in the APR values of the five cities was also found, which indicates a decrease in number of air pollution days. It suggested that Guangzhou consistently had the lowest APR value and also went through the most distinct decrease in APR—from 9% in 2000 to 1% in 2012. As indicated in Figure 3c, CAPR shows a downward trend as well, indicating the alleviation of continuous air pollution processes. In 2000, Tianjin maintained the highest CAPR value (at 34%), however, by the year 2012, Beijing had become the city with the highest CAPR (at 11%). Moreover, Shanghai and Guangzhou are shown to have experienced the most distinct decrease in CAPR and there were no continuous air pollution processes in these two cites by the year 2012.

### 4.2. Analysis of Changing Urban Form

**Figure 4:**
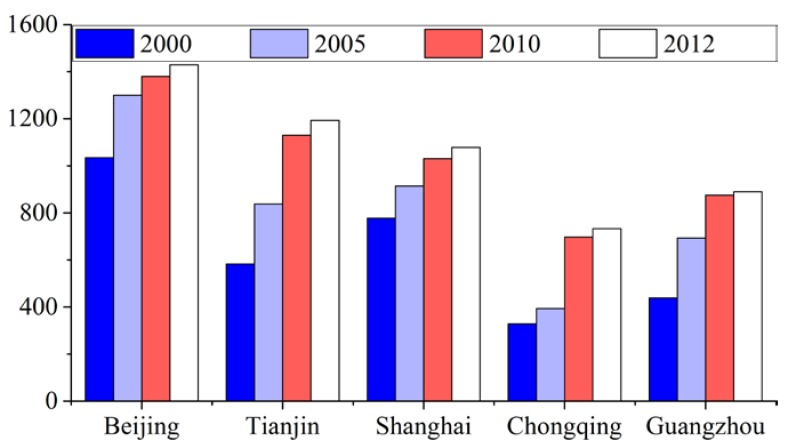
Urban areas of five Chinese megacities (km^2^), 2000–2012.

As the most rapidly developing regions in China, the five megacities addressed in this study attracted migrants from all over the country, aggregated economic activities, and thus experienced significant increases in urban size. Figure 4 portrays changes in the urban areas of the five cities over the study area, from which it can be concluded that the total urban area of each city grew rapidly between 2000 and 2012. Beijing has consistently had the largest urban area and experienced an increase of 393.74 km^2^ during the period studied, while the urban areas of Chongqing had always been the smallest despite increasing by 404.78 km^2^. Tianjin experienced the greatest urban expansion (609.99 km^2^), while Shanghai went through the least expansion (300.28 km^2^).

**Figure 5:**
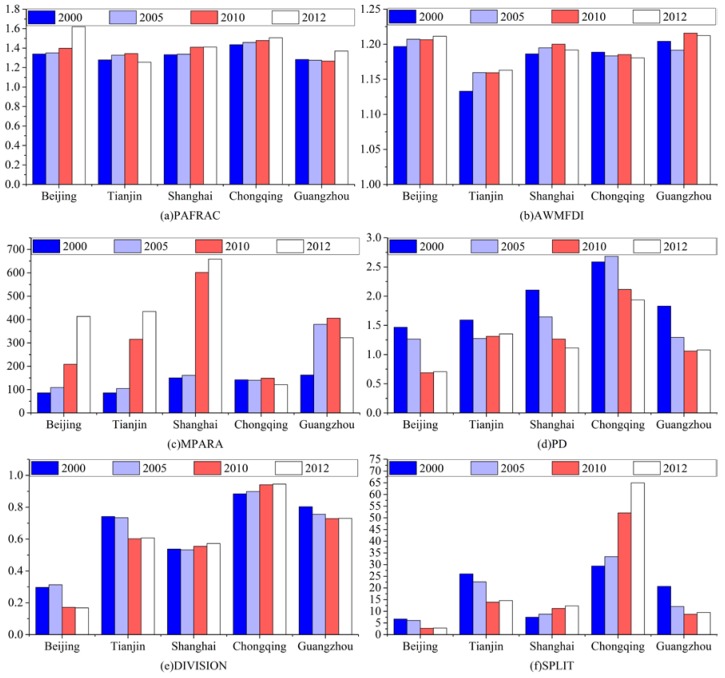
Changes in landscape metrics in five Chinese megacities, 2000–2012: (**a**) PAFRAC; (**b**) AWMFDI; (**c**) MPARA; (**d**) PD; (**e**) DIVISION; (**f**) SPLIT.

As indicated in Figure 5, the results from calculating urban shape irregularity and urban fragmentation show significant differences between cities in terms of the magnitudes and variation tendencies of the other six landscape metrics. In general, the values of PAFRAC, AWMFDI, and MPARA increase as the shape of an urban built-up area becomes more convoluted, and the values of PD, DIVISION, and SPLIT increase as the urban form becomes decentralized.

### 4.3. Estimation Results of Panel Data Models

Before performing the panel data analysis, a multicollinearity test was conducted to see whether multicollinearity, a situation in which explanatory variables are linearly correlated in a multiple regression model, existed among the seven landscape metrics selected in this study. From the test results set out in Table 4, it can be concluded that no serious multicollinearity existed among the seven independent variables. Given this, parameter estimations of the panel data models could be conducted.

In order to better understand the associations between urban form and air pollution, nine regression models were established (here referred to as Model 1 to Model 9). The dependent variables in Model 1–3, Model 4–6, and Model 7–9, are MAPI, APR, and CAPR, respectively. Among the nine models, Model 1, Model 4, and Model 7 first reviewed the regression results of TA. PAFRAC, AWMFDI, and MPARA were then added in Model 2, Model 5 and Model 8, and PD, DIVISION, and SPLIT were sequentially added in Model 3, Model 6 and Model 9. Based on the above procedure, the robustness and the reliability of the estimation results could be tested by comparing the coefficients, which indicated that the estimation results were robust and reliable. 

*F*-tests were then performed to discern which specific regression form should be used for these nine models. The results of the *F*-tests are presented in Table 5. For Model 1, given the significance level of 5%, *F*_2_ is equal to 17.51, which is larger than *F*_0.05_(1,14), indicating that *H*_2_ was rejected. Moreover, *F*_1_ is equal to 23.10, which is larger than *F*_0.05_(4,14) at the significance of 5% level; thus, *H*_1_ was rejected too, indicating that Equation (8) is the suitable form of Model 1. Similar conclusions were also drawn from the *F*-tests results for Model 2 to Model 9; thus, Equation (8) was also employed for the other eight models.

After determining the specific regression forms of these nine panel data models utilizing *F*-tests, Hausman tests—which focus on whether the generalized least squares (GLS) estimates and covariance estimator of the common parameter are obviously different, and for which the null hypothesis was that the random effects model is preferable [56]—were implemented to determine whether the fixed effects estimator or the random effects estimator should be employed for the nine panel data models. As shown in Table 6, the *p* values of the nine models were all less than the critical value at the 5% level of significance, rejecting the null hypothesis, which meant that the fixed effects model, rather than the random effects model, should be used.

Table 7, Table 8 and Table 9 exhibit the parameter estimation results of the MAPI model, APR model, and CAPR model, respectively. Surprisingly, TA is found to be negatively correlated with MAPI, APR, and CAPR, implying that urban expansion may alleviate air pollution to some extent. In the context of European and American cities, previous studies have shown that vegetation [57,58] and water [59,60] deposit and absorb air pollutants, especially particulate matter; it is for this reason that reductions of vegetation and water caused by urban expansion decrease the deposition and absorption of air pollutants and consequently aggregate air pollution. However, these studies were only undertaken from the perspective of the absorption of air pollutants without taking the emissions of air pollutants into account, and it is on the basis of this omission that a possible explanation might be offered for why urban expansion might improve air quality. It is commonly known that urban growth in Chinese cities is accompanied with the identity conversion of rural residents to city dwellers, which leads to significant lifestyle changes, including changes in household energy use. Specifically, city dwellers mostly use clean energy such as liquefied petroleum gas, natural gas, and electricity, while the direct combustion of firewood and coal makes up a considerable proportion of rural residents’ household energy consumption, especially for winter heating. The rapid urbanization process in China results in more urban population and less rural population [61]. The substitution of clean energy for dirty energy resulting from urbanization in China can thus contribute significantly to improvements in air quality. Furthermore, it suggested that PAFRAC, AWMFDI, and MPARA displayed positive impacts in relation to APR, indicating that complex urban shape helps to increase the number of air pollution days, which is accompanied by detrimental health effects. Exhaust emission from household vehicle travel, which has a strong correlation with urban form [62], is a main source of air pollution in many cities [63]. High urban shape irregularity may result in disorder and chaos in traffic, with the subsequent traffic congestions being characterized by low vehicle speeds, long vehicle trips, and a large volume of air pollutant emissions [4]. Due to the temporal characteristics of citizens’ travel behavior, significant daily differences exist in the degrees of traffic congestion and consequently of air pollution. As such, regular urban geometry is conducive to reducing APR, which takes the daily differences of air pollution into account. In addition, PD, DIVISION, and SPLIT demonstrated significant negative correlations with CAPR. In the context of European and American cities, numerous previous studies have concluded that fragmented urban form can worsen the job-housing imbalance, which leads to a more auto-dependent lifestyle characterized by higher single-occupant vehicles use and larger vehicle kilometers of travel (VKT), and thus aggravates air pollution [64]. However, compact urban form was found to be conductive to the persistence of air pollutants and thus led to continuous air pollution processes in five Chinese cities, which results in a contrasting conclusion. This finding can be ascribed to the effects of urban form on microclimate in cities, through qualities such as temperature and air motion. Temperature is influenced by urban form mainly through the heat island effect, which can be aggravated by continuous impervious surfaces in cities [65,66]. Hence, compact and continuous urban form results in rising temperatures, which help to accumulate air pollutants. Stagnant atmospheric conditions prevent the dispersal of air pollutants [4], as mass skyscrapers constitute obstacles to air flow and reduce wind speed [67]. Moreover, it should also be noted that compact urban form with high-density city centrality may increase exposure to air pollution by gathering population, which consequently would result in severe adverse health effects.

Based on this study, it might prove fruitful to further explore how urban form affects air pollution in Chinese cities, and some study prospects are proposed as follows. Firstly, relevant studies are expected to be conducted at a smaller level, such as at the land parcel level. This research topic is likely to be better addressed at a smaller level, as the effects of urban form on air pollution may vary across land parcels. Secondly, more Chinese cities are expected to be incorporated, in order to examine the diverse impacts of urban form on air pollution in cities with different characteristics. Last but not least, sensitivity analysis is expected to be applied in relevant studies, particularly global sensitivity and uncertainty analyses (GSUA) [68,69], a variance-based method for analyzing data and models given an objective function [70]. GSUA can highlight the uncertainty-sensitivity-complexity of the model/data as well as management implications of the model and/or results [71]. In addition, the interrelatedness among selected variables can be assessed using maximum entropy networks (MENets) [72].

## 5. Conclusions

Air pollution has emerged as a prominent threat to global sustainability. Much scholarly attention has been directed towards air pollution from a range of perspectives, and urban form is increasingly being identified as a key determinant of air pollution in developed countries. However, a limited number of studies addressed the impacts of urban form on air pollution in developing countries, especially in China, the world’s largest developing country [73]. In order to fill this gap, this study estimated the effects of urban form on air pollution, focusing on the five most rapidly-developing megacities in China.

On the basis of API, three quantitative indicators (i.e., MAPI, APR, and CAPR) were developed to evaluate different aspects of air pollution in five Chinese megacities. A range of landscape metrics, which characterized three aspects of urban form (i.e., urban size, urban shape irregularity, and urban fragmentation), were then calculated based on remote sensing data. Panel data modeling was subsequently performed in order to estimate the associations between urban form and air pollution in the five Chinese cities.

The results suggested that TA exerted negative impacts on MAPI, APR, and CAPR, indicating that urban expansion helped to abate air pollution, a finding that is inconsistent with previous studies addressing European and American cities. The substitution of clean energy for dirty energy that results from urbanization in China provided a possible explanation for this finding. Moreover, PAFRAC, AWMFDI, and MPARA were found to positively correlate with APR, which suggested that irregular urban shape contributed to increasing the number of air pollution days. As APR is an indicator that takes daily differences in air pollution into account and the traffic congestion arising from high urban shape complexity has temporal characteristics as well, which leads to more air pollutants emissions, the geometry of urban land was believed to affect the number of air pollution days by influencing traffic configuration in Chinese cities. In addition, PD, DIVISION, and SPLIT were found to negatively correlate with CAPR, a result that indicated that compact urban form could aggravate continuous air pollution processes. This finding was attributed to the effects of compact urban form on microclimate in Chinese cities, through the urban heat island effect and stagnant atmospheric conditions that prevent the dispersal of air pollutants.

The findings obtained from this study hold a range of implications for policy making. Firstly, the disordered urban sprawl should be controlled, and sustainable urban growth and moderate urban sizes should be advocated alternatively. For example, transit-oriented development, which encourages more biking, walking, and public transit use [74], can reduce auto-dependency and consequent air pollution. Only in this way can the benefits of urban expansion be preserved while its potential negative impacts are reduced. Moreover, the geometry of urban land should be optimized to lower irregularity, as urban form with low shape irregularity not only reduces vehicle travel demand, but also increases road density and street accessibility, both of which are associated with less traffic congestion, more efficient transportation systems, and consequent reduction of mobile-source emissions. Last but not the least, since the decentralization of urban functions and polycentric urban form are of great significance for facilitating the dispersal of air pollutants and terminating the persistence of air pollution, “leapfrog” development strategy and decentralized spatial configurations should be advocated to shape polycentric urban form rather than monocentric urban form, and to conserve greenbelt.

## Figures and Tables

**Table 1:** Statistical characteristics of Air pollution index (API) in China.

API ^1^	Air Quality Class	Air Quality Evaluation	Health Effects
0–50	I	Excellent	Harmless
51–100	II	Good	Acceptable
101–200	III	Mild pollution	Unhealthy for sensitive population
201–300	IV	Moderate pollution	Unhealthy
301–500	V	Severe pollution	Very unhealthy

^1^ Air pollution index.

**Table 2:** Intervals for pollutant concentrations and corresponding API.

Intervals for Pollutant Concentrations (mg/m^3^)			API Intervals
SO_2_^1^	NO_2_^2^	PM_10_^3^	
[0.000, 0.050]	[0.000, 0.080]	[0.000, 0.050]	0–50
(0.050, 0.150]	(0.080, 0.120]	(0.050, 0.150]	51–100
(0.150, 0.800]	(0.120, 0.280]	(0.150, 0.350]	101–200
(0.800, 1.600]	(0.280, 0.565]	(0.350, 0.420]	201–300
(1.600, 2.100]	(0.565, 0.750]	(0.420, 0.500]	301–400
(2.100, 2.620]	(0.750, 0.940]	(0.500, 0.600]	401–500

^1^ Sulfur dioxide; ^2^ Nitrogen dioxide; ^3^ Particulate matter with particle size less than 10 mu.

**Table 3:** Description of landscape metrics used in the study.

Landscape Metric	Equation
Total landscape area (TA)	$\;\mathrm{TA}=\overset{n}{\underset{j=1}{\sum }}a_{ij}\left(1/10000\right)$
Perimeter-area fractal dimension (PAFRAC)	$\;\mathrm{PAFRAC}=\frac{\frac{2}{\left[N\sum_{i=1}^{m}\sum_{j=1}^{n}\left(\ln p_{ij}\cdot \ln a_{ij}\right)\right]-\left[\left(\sum_{i=1}^{m}\sum_{j=1}^{n}\ln p_{ij}\right)\left(\sum_{i=1}^{m}\sum_{j=1}^{n}\ln a_{ij}\right)\right]}}{\left(N\sum_{i=1}^{m}\sum_{j=1}^{n}\ln p_{ij}{}^{2}\right)-{\left(\sum_{i=1}^{m}\sum_{j=1}^{n}\ln p_{ij}\right)}^{2}}$
Area-weighted mean fractal dimension index (AWMFDI)	$\;\mathrm{AWMFDI}=\overset{m}{\underset{i=1}{\sum }}\overset{n}{\underset{j=1}{\sum }}\left[\left(\frac{2\ln\left(0.25p_{ij}\right)}{\ln\left(a_{ij}\right)}\right)\left(\frac{a_{ij}}{A}\right)\right]$
Mean perimeter-area ratio (MPARA)	$\;\mathrm{MPARA}=\frac{\sum_{i=1}^{m}\sum_{j=1}^{n}\left(p_{ij}/a_{ij}\right)}{mn}$
Patch density (PD)	$\;\mathrm{PD}=\frac{N}{A}$
Landscape division index (DIVISION)	$\;\mathrm{DIVISION}=1-\overset{m}{\underset{i=1}{\sum }}\overset{n}{\underset{j=1}{\sum }}{\left(\frac{a_{ij}}{A}\right)}^{2}$
Splitting index (SPLIT)	$\;\mathrm{SPLIT}=\frac{A^{2}}{\sum_{i=1}^{m}\sum_{j=1}^{n}a_{ij}{}^{2}}$

*a_ij_*= area of patch *ij*; *N* = total number of patches in the landscape; *p_ij_*= perimeter of patch *ij*; *A* = total landscape area.

**Table 4:** Pearson’s correlation coefficients of the independent variables.

	TA	PAFRAC	AWMFDI	MPARA	PD	DIVISION	SPLIT
TA	1	-	-	-	-	-	-
PAFRAC	−0.627 ***	1	-	-	-	-	-
AWMFDI	0.257	0.036	1	-	-	-	-
MPARA	0.409 *	−0.698 ***	0.249	1	-	-	-
PD	−0.679 ***	0.737 ***	−0.129	−0.223	1	-	-
DIVISION	−0.768 ***	0.434 *	−0.332	−0.144	0.779 ***	1	-
SPLIT	−0.498 **	0.645 ***	−0.163	−0.304	0.793 ***	0.782 ***	1

*** *p* < 0.01; ** *p* < 0.05; * *p* < 0.1.

**Table 5:** *F*-test results.

	Hypothesis *H*_2_	Hypothesis *H*_1_
Model 1	*F*_0.05_(1,14) < 17.51	*F*_0.05_(4,14) < 23.10
Model 2	*F*_0.05_(4,11) < 10.17	*F*_0.05_(4,11) < 13.74
Model 3	*F*_0.05_(7,8) < 8.15	*F*_0.05_(4,8) < 7.34
Model 4	*F*_0.05_(1,14) < 6.26	*F*_0.05_(4,14) < 16.88
Model 5	*F*_0.05_(4,11) < 2.17	*F*_0.05_(4,11) < 6.83
Model 6	*F*_0.05_(7,8) < 7.23	*F*_0.05_(4,8) < 6.74
Model 7	*F*_0.05_(1,14) < 11.87	*F*_0.05_(4,14) < 16.84
Model 8	*F*_0.05_(4,11) < 3.69	*F*_0.05_(4,11) < 5.59
Model 9	*F*_0.05_(7,8) < 7.10	*F*_0.05_(4,8) < 5.25

**Table 6:** Hausman test results.

	Chi-Sq Statistic	*p-*Values	Type of Regression Model
Model 1	6.95	0.0004	Fixed effects
Model 2	10.72	0.0015	Fixed effects
Model 3	13.14	0.0121	Fixed effects
Model 4	12.58	0.0007	Fixed effects
Model 5	15.63	0.0023	Fixed effects
Model 6	18.40	0.0103	Fixed effects
Model 7	8.82	0.0007	Fixed effects
Model 8	14.37	0.0002	Fixed effects
Model 9	17.23	0.0160	Fixed effects

**Table 7:** Estimation results for MAPI model.

Independent Variables	MAPI Model		
	Model 1	Model 2	Model 3
ln TA	−0.300 ***	−0.285 ***	−5.401 ***
	(0.0717)	(0.102)	(14.71)
ln PAFRAC	-	−2.887	−233.0 *
	-	(1.675)	(132.2)
ln AWMFDI	-	−1.771	−158.0
	-	(3.536)	(148.5)
ln MPARA	-	−0.155 *	−24.77 *
	-	(0.0747)	(7.886)
ln PD	-	-	156.2 *
	-	-	(62.16)
ln DIVISION	-	-	−78.20 *
	-	-	(37.25)
ln SPLIT	-	-	0.669
	-	-	(1.300)
R-squared ^1^	0.781	0.835	0.877

*** *p* < 0.01; ** *p* < 0.05; * *p* < 0.1; ^1^ Coefficient of determination.

**Table 8:** Estimation results for APR model.

Independent Variables	APR Model		
	Model 4	Model 5	Model 6
ln TA	−1.107 **	−1.181 **	−0.208 **
	(0.443)	(0.816)	(0.152)
ln PAFRAC	-	5.616 ***	2.898 ***
	-	(13.43)	(1.125)
ln AWMFDI	-	23.70 **	1.348 ***
	-	(28.36)	(3.066)
ln MPARA	-	0.0431 **	0.168 **
	-	(0.599)	(0.0599)
ln PD	-	-	−0.531
	-	-	(0.653)
ln DIVISION	-	-	−0.110 *
	-	-	(0.375)
ln SPLIT	-	-	−0.0252
	-	-	(0.0148)
R-squared ^1^	0.802	0.841	0.864

*** *p* < 0.01; ** *p* < 0.05; * *p* < 0.1; ^1^ Coefficient of determination.

**Table 9:** Estimation results for CAPR model.

Independent Variables	CAPR Model		
	Model 7	Model 8	Model 9
ln TA	−1.743 ***	−1.688 ***	−0.242 ***
	(0.506)	(0.921)	(0.140)
ln PAFRAC	-	−14.15	−0.570 *
	-	(15.17)	(1.029)
ln AWMFDI	-	−0.411	−0.0958
	-	(32.02)	(2.806)
ln MPARA	-	−0.821	−0.0192
	-	(0.677)	(0.0548)
ln PD	-	-	−2.733 ***
	-	-	(0.598)
ln DIVISION	-	-	−0.745 **
	-	-	(0.343)
ln SPLIT	-	-	−0.165 ***
	-	-	(0.0136)
R-squared ^1^	0.796	0.827	0.861

*** *p* < 0.01; ** *p* < 0.05; * *p* < 0.1; ^1^ Coefficient of determination.
